# The Rate of Underascertainment of Novel Coronavirus (2019-nCoV) Infection: Estimation Using Japanese Passengers Data on Evacuation Flights

**DOI:** 10.3390/jcm9020419

**Published:** 2020-02-04

**Authors:** Hiroshi Nishiura, Tetsuro Kobayashi, Yichi Yang, Katsuma Hayashi, Takeshi Miyama, Ryo Kinoshita, Natalie M. Linton, Sung-mok Jung, Baoyin Yuan, Ayako Suzuki, Andrei R. Akhmetzhanov

**Affiliations:** 1 Graduate School of Medicine, Hokkaido University, Kita 15 Jo Nishi 7 Chome, Kita-ku, Sapporo-shi, Hokkaido 060-8638, Japan; tootsieroll2910@gmail.com (T.K.); lukeyang1993@eis.hokudai.ac.jp (Y.Y.); katsuma5miffy@gmail.com (K.H.); ryokinoshita@med.hokudai.ac.jp (R.K.); nlinton@gmail.com (N.M.L.); seductmd@med.hokudai.ac.jp (S.-m.J.); baoyinyuan@outlook.com (B.Y.); akmskorokoro@gmail.com (A.S.);; 2 Core Research for Evolutionary Science and Technology, Japan Science and Technology Agency, Honcho 4-1-8, Kawaguchi, Saitama 332-0012, Japan; 3 Osaka Institute of Public Health, Nakamichi 1-3-69, Higashinari, Osaka 537-0025, Japan; takeshi.j.miyama@gmail.com

**Keywords:** epidemiology, ascertainment, diagnosis, travel, importation, statistical inference

## Abstract

From 29 to 31 January 2020, a total of 565 Japanese citizens were evacuated from Wuhan, China on three chartered flights. All passengers were screened upon arrival in Japan for symptoms consistent with novel coronavirus (2019-nCoV) infection and tested for presence of the virus. Assuming that the mean detection window of the virus can be informed by the mean serial interval (estimated at 7.5 days), the ascertainment rate of infection was estimated at 9.2% (95% confidence interval: 5.0, 20.0). This indicates that the incidence of infection in Wuhan can be estimated at 20,767 infected individuals, including those with asymptomatic and mildly symptomatic infections. The infection fatality risk (IFR)—the actual risk of death among all infected individuals—is therefore 0.3% to 0.6%, which may be comparable to Asian influenza pandemic of 1957–1958.

## 1. Introduction

As the epidemic of novel coronavirus (2019-nCoV) unfolded, the government of China has decided to implement countainment measures, including discontinuation of ordinary flights to and from Wuhan Tianhe International Airport even during the Chinese New Year holidays. The impact of such a dramatic restriction on human migration on the epidemic dynamics of 2019-nCoV will be soon visually identifiable, but this change made it difficult to update the cumulative incidence estimate using exported case data outside China [1]. Presently, exponential growth of confirmed cases has continued in China, but a substantial number of infections may have missed being diagnosed and reported.

In the face of travel restrictions to and from Wuhan, the government of Japan offered Japanese citizens in Wuhan the option to be evacuated on specially chartered flights from 29 to 31 January 2020. A total of 565 Japanese citizens boarded three different flights and were evacuated. As of 2 February 2020, healthy individuals are quarantined with movement restrictions and brought under medical observation at designated hotels and dormitories. An additional 63 individuals who were determined to have symptoms consistent with 2019-nCoV infection upon arrival in Japan were all admitted to hospitals. The evacuation procedures involved in these flights have offered a unique opportunity for evaluation of the rate of underascertainment of 2019-nCoV cases in China. Here, we report the estimated rate of underascertainment and an update on current incidence in Wuhan.

## 2. Statistical Estimation

There were two interesting features of the evacuation process conducted by Japanese authorities. First, all 565 passengers were screened for symptoms upon arrival. Prior to disembarkation, the quarantine officers used portable thermoscanners to screen the temperature. In the following, all passengers were interviewed regarding whether they have any symptoms suggestive of upper raspiratory tract infection, including fever and cough. Of the 565 passengers, 63 were symptomatic. Second, the passengers were tested for presence of 2019-nCoV using reverse transcription polymerase chain reaction (RT-PCR), and eight passengers (1.4%) were determined to have the virus. Importantly, most of the individuals positive for the virus were asymptomatic (five passengers), while only the other three had symptoms consistent with 2019-nCoV infection, indicating that the dataset generated from this process can help investigate the full spectrum of infection including mild and asymptomatic infections. 

Let *n* and *c*(*t*) be the population size of Wuhan (11 million) and the cumulative number of cases in Wuhan by time *t*. As of 29 January 2020, there were 1905 confirmed cases reported from Wuhan. Suppose that *q* is the fraction of underascertained infected individuals and *T* is the detection window for the virus. While *T* is assumed as 10.0 days elsewhere [2] and even longer in an earlier study [1], the mean serial interval has recently been estimated at 7.5 days [3]. Another modeling study estimated the mean infectious period to be 3.6 days [4]. Here we set the serial interval to be 7.5 days as the default, and 3.6 and 10.0 days as the possible range of uncertainty. The balance equation for the risk of infection is thus written:

(1) $$\frac{8}{565}=\frac{c\left(t\right)T}{qn}$$

It should be noted that all Japanese evacuees resided in Wuhan City, and citizens living in other cities in Hubei were not included on the flights. Hence, the population size *n* is not the catchment population of the Wuhan airport but the population size of Wuhan City. The likelihood function to estimate *q* is:

(2) $$L\left(q;8\;\mathrm{infections}\;\mathrm{out}\;\mathrm{of}\;565\;\mathrm{passengers}\right)=\left(\begin{array}{c} 565\\ 8 \end{array}\right){\left(\frac{c\left(t\right)T}{qn}\right)}^{8}{\left(1-\frac{c\left(t\right)T}{qn}\right)}^{557}$$

Subsequently, the cumulative incidence can be estimated by: $c\left(t\right)/\hat{q}$.

## 3. Estimate of the Incidence of “Infection”

Assuming that the mean detection window of the virus can be informed by the mean serial interval, here set at 7.5 days [3] (Table 1), the ascertainment rate was estimated to be 9.2% (95% confidence interval [CI]: 5.0, 20.0). Allowing the serial interval to vary from 3.6 to 10.0 days, the ascertainment rate ranged from 4.4% (95% CI: 2.4, 9.6) to 12.2% (95% CI: 6.6, 26.7). An ascertainment rate of 9.2% indicates that there are approximately 20,767 infected individuals (95% CI: 9528, 38,421) in Wuhan City as of 29 January 2020. 

Laboratory testing of all evacuated passengers upon arrival in Japan offered an important opportunity to explicitly quantify the ascertainment rate using the number of both symptomatic ana asymptomatic infected evacuees for the calculatoin. In other words, if we assume that about 9.2% (95% CI: 5.0, 20.0) of cases have been diagnosed and reported by the Chinese government, the remaining 90.8% (80.0% or 95.0%) did not result in notification. This could be due to a variety of reasons including asymptomatic infection, mild illness, underdiagnosis and underreporting.

The derived estimate is key in interpreting the severity of the 2019-nCoV infection. Presently, the case fatality risk among confirmed cases (cCFR) has been estimated to be in the order of 3%–6% [5]. However, the risk of death among all infected individuals is likely much smaller. With our estimate of ~10% ascertainment, the actual risk of death is therefore 0.3% to 0.6% among all infected individuals (abbreviated as iCFR or IFR) [6], which may be comparable to the Asian influenza pandemic of 1957–1958. However, it should be noted that quoted values of IFR may be oversestimated if the ascertainment rate is biased due to difficulties in detection of 2019-nCoV using RT-PCR (e.g., very short detection window of the virus during the course of infection). 

Clarifying the full spectrum of disease is of utmost importance to understanding the expected burden of the ongoing epidemic to the world. The meticulous effort of screening and testing Japanese passengers on evacuation flights indicated that about 90% of infections were not initially ascertained as cases, which must be kept in mind when analyzing the confirmed case data in China.

## Figures and Tables

**Table 1:** Exportation events and estimated incidence in China.

Detection Window (Days)	Ascertainment Rate (%)	Incidence in Wuhan as on 29 January 2020 (Persons)
3.6	4.4 (2.4, 9.6)	43,265 (19,849, 80,043)
7.5	9.2 (5.0, 20.0)	20,767 (9528, 38,421)
10.0	12.2 (6.6, 26.7)	15,575 (7146, 28,816)

The 95% confidence interval derived from profile likelihood is given in the parentheses. The estimated incidence represents infection, inclusive of mild and asymptomatic ones.
